# Biomechanical analysis of T10-T11 vertebrae under the combined effects of osteoporosis and L4 spondylolisthesis: a finite element analysis based on computed tomography

**DOI:** 10.3389/fbioe.2026.1897265

**Published:** 2026-09-29

**Authors:** Zhizhou Yang, Xiaowei Lian, Yunsheng Wang, Xiao Xiong, Zimo Wang, Linfeng Wang, Xiaobin Wang, Feng Wang

**Affiliations:** 1 Department of Spinal Surgery, Third Hospital of Hebei Medical University, Shijiazhuang, China; 2 Department of Spinal Surgery, The Second Xiangya Hospital of Central South University, Changsha, China

**Keywords:** biomechanics, finite element analysis, image reconstruction, osteoporosis, spondylolisthesis, synergistic effect

## Abstract

**Aims:**

Osteoporotic vertebral compression fractures are common; however, the biomechanical link between L4 spondylolisthesis and non-adjacent thoracic fractures remains unclear. This study used 3D finite element (FE) analysis to assess the biomechanical effects of L4 spondylolisthesis and osteoporosis on T10–T11.

**Methods:**

Four FE models of T10–L5 were reconstructed from CT images of a healthy adult male: normal (A), isolated spondylolisthesis (B), isolated osteoporosis (C), and combined (D). A 500-N axial load and 10-N·m moments simulated six motions. Maximum von Mises stresses of whole vertebra and cancellous bone, and segmental ROM at T10–T11, were compared.

**Results:**

L4 spondylolisthesis alone increased T10–T11 ROM (4.5%–24.7%) and stress versus normal. Osteoporosis alone markedly raised T10 overall stress (+73.9%) and cancellous stress (+19.6%) during flexion. The combined model showed the worst deterioration: peak overall stress 59.07 MPa (flexion) and cancellous stress 1.06 MPa (lateral bending). Stress nephograms confirmed concentration at the anterocentral body and pedicles, with wider distribution and higher magnitudes in pathological models. The isolated spondylolisthesis effect on stress (0.4%–15.1%) was far smaller than osteoporosis alone, and combined elevation was predominantly driven by osteoporosis. Yet synergy was most pronounced in cancellous bone—the primary load-bearing structure—where combined effects exceeded the sum of individual ones by up to 7.6 percentage points, highlighting spondylolisthesis as a critical amplifier of fracture-relevant stress in osteoporotic vertebrae.

**Conclusion:**

L4 spondylolisthesis moderately increases thoracic stress and ROM, but osteoporosis remains the main determinant of T10–T11 stress. Spondylolisthesis acts as a secondary modifier that synergistically amplifies cancellous bone stress beyond additive expectations once osteoporosis compromises vertebral strength. This synergy is the key biomechanical link between lumbar spondylolisthesis and the clinically observed rise in thoracic fracture risk. Osteoporosis worsens this effect, deteriorating the upper thoracic biomechanical environment and increasing susceptibility to secondary fractures. Clinically, concomitant lumbar spondylolisthesis in osteoporotic patients should be recognized as a key biomechanical marker for thoracic fracture risk.

## 1 Introduction

Osteoporosis (OP) is a systemic skeletal disease characterized by reduced bone mass and deterioration of bone microarchitecture, leading to increased bone fragility and fracture risk (Compston et al., 2019). Osteoporotic vertebral compression fractures (OVCFs) are one of the most common and serious complications of osteoporosis, often causing severe pain, kyphotic deformity, and significantly impairing patients‘ quality of life and mobility (Choi et al., 2022; Hwang et al., 2023; Lee et al., 2024). Therefore, early identification of risk factors for OVCFs is crucial to reduce incidence. Although low bone density is the fundamental cause, epidemiological studies have shown that only a subset of osteoporotic patients develop symptomatic fractures, suggesting that other important biomechanical factors besides bone density are involved (Lee and Bae, 2023). The occurrence of OVCFs reflects a failure of skeletal adaptation, and its pathophysiology involves a combination of unbalanced bone remodeling, microarchitectural deterioration, and biomechanical factors.

Lumbar spondylolisthesis (LS) is defined as partial or complete slippage of one vertebra relative to the adjacent inferior vertebra due to congenital dysplasia, trauma, strain, or degenerative changes causing abnormal bony connection. It is a major cause of lower back pain in adults and a common condition in spinal surgery. Clinically, the overall incidence of LS in the general population is about 4%–8.7%, with anterior slippage of L4 and L5 being the most common (Fredrickson et al., 1984; Jacobsen et al., 2007). It is reported that only about 10% of LS patients require surgical treatment. In this study, the term “thoracolumbar” refers to the transitional region between the thoracic and lumbar spine, encompassing the lower thoracic vertebrae (T10-T12) and the upper lumbar segments. Studies have found that patients with thoracolumbar OVCFs often have concurrent LS, with an incidence of about 10.1% (Wang et al., 2022); retrospective studies have reported that LS is an independent risk factor for thoracolumbar OVCFs (Wang et al., 2022; Wang et al., 2018), providing new insights for prevention, diagnosis, and treatment of OVCFs, and offering a new basis for fully understanding LS and its potential risks. Previous studies have found that spinopelvic parameters are significantly altered in LS patients, with increased thoracolumbar kyphosis and thoracic kyphosis, especially in isthmic spondylolisthesis. These alterations of spinopelvic alignment indicate that the mechanical consequences of LS are not confined to the lumbar spine but may be transmitted along the spinal column to the thoracolumbar region. Huang et al. found that spinal kyphosis itself is an important risk factor for future OVCFs, independent of low bone density or fracture history (Huang et al., 2006). However, the specific biomechanical mechanism by which LS induces OVCFs remains unclear. Notably, our previous radiographic and clinical correlation studies have preliminarily confirmed a significant association between LS and thoracolumbar OVCFs (Wang et al., 2022; Wang et al., 2018); the present study was therefore designed to elucidate the biomechanical mechanism underlying this clinical observation.

Finite element analysis provides a powerful tool for studying such complex, multi-segment spinal biomechanical problems. It allows precise quantification of the impact of specific pathological changes on stress and strain distribution in spinal structures under controlled conditions (Zhang et al., 2024). Therefore, this study aims to systematically analyze the biomechanical effects of L4 spondylolisthesis and osteoporosis, alone and in combination, on the thoracolumbar spine, as well as imaging changes caused by spondylolisthesis, using three-dimensional finite element models of the thoracolumbar spine and related imaging data, thereby elucidating the mechanical mechanism underlying the hypothesis that lumbar spondylolisthesis may increase the risk of thoracic vertebral fractures.

## 2 Methods

### 2.1 CT image segmentation and reconstruction

This study was approved by the ethics committee and informed consent was obtained from the patient. Continuous thin-slice CT scan data of the thoracolumbar spine were obtained from a healthy male volunteer. The DICOM images of the T10-L5 segment were imported into medical image processing software Mimics 21.0 (Materialise, Belgium). Through threshold segmentation, region growing, and manual editing, three-dimensional masks of the bony structures of each vertebra from T10 to L5 were extracted, and preliminary surface triangular mesh models were generated. The models were then imported into reverse engineering software Geomagic 2021 (Geomagic, United States) for surface optimization, including noise removal and smoothing, to finally obtain the three-dimensional geometric model of the T10-L5 segment. After smoothing, the model was duplicated, and the duplicate was renamed “cancellous bone”. An inward offset of 2 mm was applied to simulate cortical bone thickness. A cortical shell thickness of 2 mm was adopted in accordance with previously validated spinal finite element models employing the same 2 mm shell constructed through a comparable Mimics-Geomagic-SolidWorks workflow (Li C. et al., 2024; Shash and Elden, 2025), and the posterior elements were removed to obtain the cancellous bone, as shown in Figure 1. The model was checked for errors and confirmed to be anatomically realistic.

**FIGURE 1:**
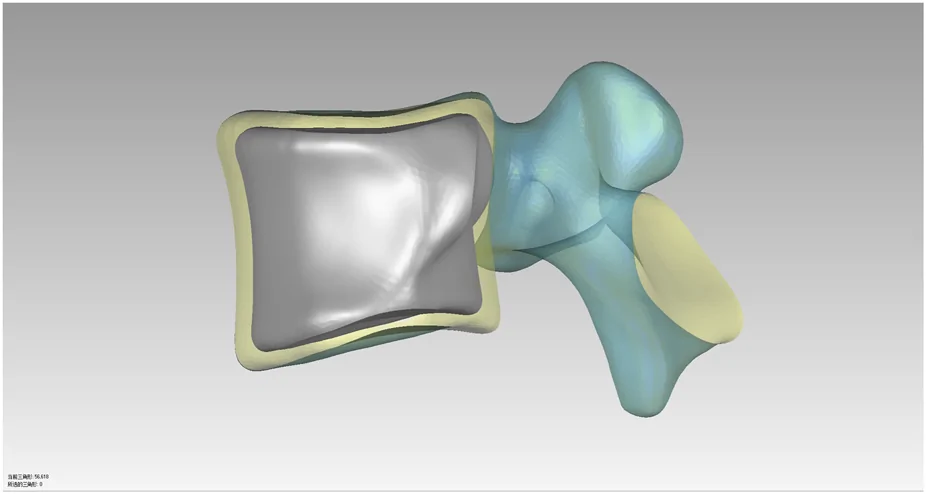
Separation of cortical and cancellous bone: The smoothed vertebral body model was duplicated, and the duplicated model was renamed “cancellous bone”. Subsequently, using the offset function of the software, the cancellous bone model was inwardly offset by 2 mm to simulate the thickness of the cortical bone. Finally, the posterior elements (e.g., pedicles and spinous process) of this model were removed, retaining only the structure representing the inner cancellous bone, to obtain the final cancellous bone model.

### 2.2 Finite element model

The geometric models were imported into SolidWorks 2021 (Dassault System, France) for assembly. First, intervertebral discs and cartilage structures were created using lofting, extrusion, and other commands. Second, four three-dimensional finite element models of T10-L5 were reconstructed by displacing the L4 vertebra anteriorly relative to L5 by 7.6 mm, corresponding to 23.8% of the 31.98 mm anteroposterior width of the L5 superior endplate. All T10-L5 models were based on CT images of normal anatomy. Model A: control group. Model B: spondylolisthesis group. The two finite element models are shown in Figure 2. The resulting models were then imported into the finite element analysis software Ansys 2021R1 (Ansys, United States) to establish numerical models and perform meshing. Table 1 lists the element counts for each model. The node and element numbers of the model meshes were determined through a mesh convergence analysis: the vertebrae were meshed with 2 mm second-order tetrahedral elements, the endplates and cartilage with 0.65 mm elements, and the annulus fibrosus and nucleus pulposus with 1 mm elements; further refinement altered the peak stresses by less than 5%, satisfying the convergence criteria. Identical meshing parameters and element types were applied to all four models to ensure comparability; the minor difference in element counts between the normal and spondylolisthesis models arises from the altered L4 geometry and re-meshing. T10 and T11 were selected as the observation segments because, as the segments most remote from the L4 lesion within this thoracolumbar model covering all thoracolumbar vertebrae, they best demonstrate the remote biomechanical effect of the slippage.

**FIGURE 2:**
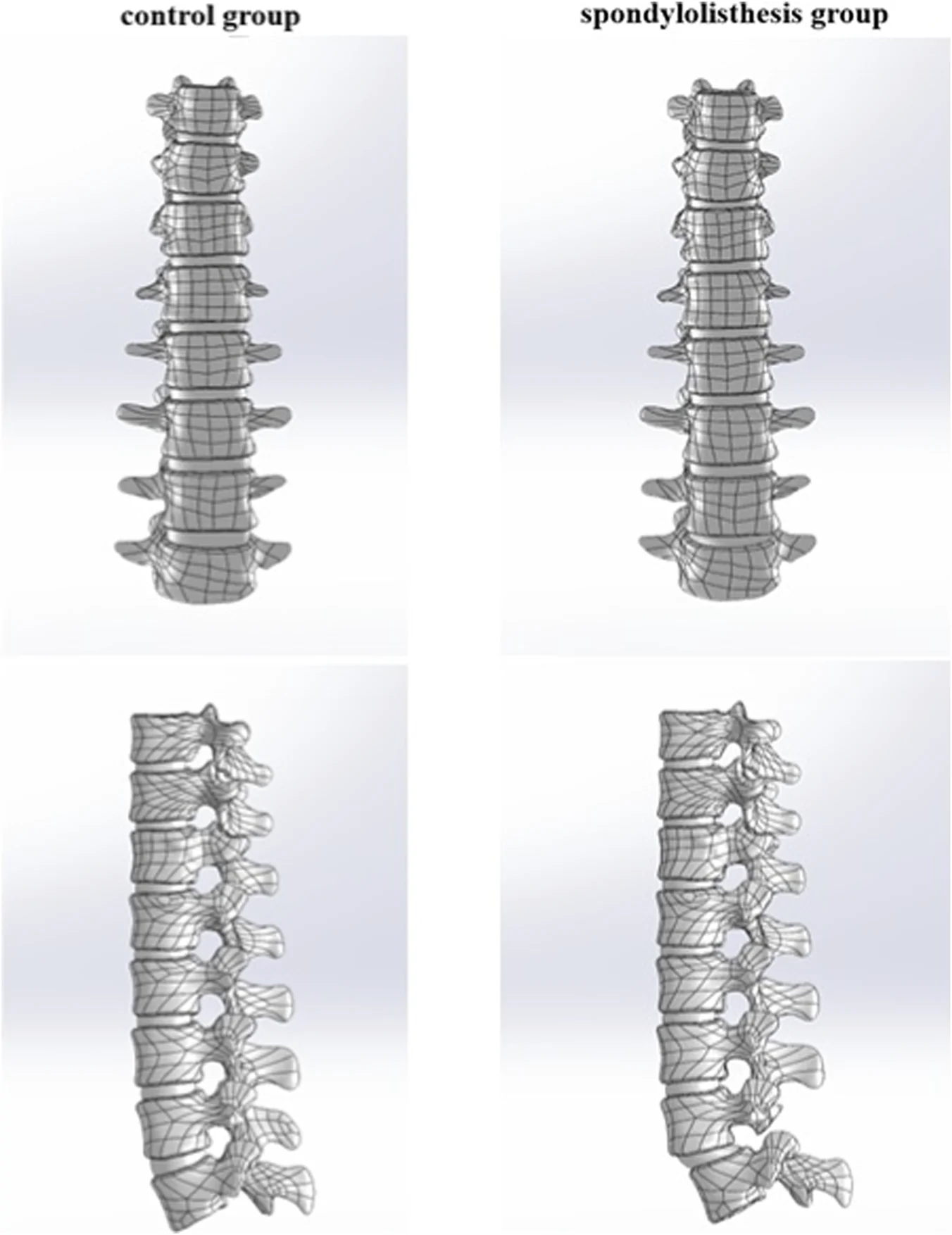
In the assembly, the cortical and cancellous bones are reset and assembled according to the origin, and the intervertebral discs, cartilage, etc. are made using commands such as layout, segmentation, and stretching in SolidWorks based on the vertebral body assembly model. Establish Model A: Control Group; Model B: Slip group as shown in the figure.

**TABLE 1:** The model detail.

Model	Model A (control)	Model B (spondylolisthesis)	Model C (osteoporosis)	Model D (osteoporosis + spondylolisthesis)
Nodes	1348312	1324012	1348312	1324012
Elements	803755	788448	803755	788448

### 2.3 Material properties

All materials were assumed to be homogeneous, isotropic, and linearly elastic. Specific material parameters are listed in Table 2, referencing previously published biomechanical experiments and finite element studies. The material properties of the osteoporotic model were scaled down from normal bone parameters: specifically, the elastic modulus of cortical bone was reduced from 12,000 MPa to 8,040 MPa and that of cancellous bone to 34 MPa, a reduction scheme consistent with previously published osteoporotic finite element models (Li et al., 2025). Important spinal ligaments (anterior longitudinal ligament, posterior longitudinal ligament, interspinous ligament, supraspinous ligament, intertransverse ligament, ligamentum flavum, and facet capsular ligament) were included, with material parameters shown in Table 3. The osteoporotic versions of Models A and B were also derived, designated Model C (osteoporosis) and Model D (spondylolisthesis + osteoporosis), respectively.

**TABLE 2:** Material parameters of bone.

Structure	Elastic modulus (MPa)	Poisson’s ratio
Cortical bone	12000	0.3
Cancellous bone	100	0.3
Endplate	25	0.3
Annulus fibrosus	4.2	0.453
Nucleus pulposus	1	0.499
Articular cartilage	50	0.3
Osteoporotic cortical bone	8,040	0.3
Osteoporotic cancellous bone	34	0.2
Nucleus pulposus (spondylolisthesis)	2	0.499
Annulus fibrosus (spondylolisthesis)	8.4	0.453

**TABLE 3:** Material parameters of Ligament.

Ligament	Stiffness (N/mm)
Anterior longitudinal ligament	8.74
Posterior longitudinal ligament	5.83
Interspinous ligament	10.85
Supraspinous ligament	2.39
Intertransverse ligament	0.19
Ligamentum flavum	15.38
Facet capsular ligament	15.75

### 2.4 Boundary and loading conditions

The same boundary and loading conditions were applied to all four models. The inferior surface of L5 was fixed. A 500 N vertical load was applied on the superior endplate of T10 through a reference node coupled to the T10 superior endplate surface (in Figure 3), together with a 10 Nm moment to simulate six physiological motions: flexion, extension, left/right lateral bending, and left/right axial rotation. The range of motion (ROM) and maximum stress of the four model groups were measured. Regarding contact definitions, bonded contact was assigned among the cortical bone, cancellous bone, and endplates within each vertebra, allowing no relative displacement; surface-to-surface contact was defined among the annulus fibrosus, nucleus pulposus, and endplates; the superior and inferior facet joints were defined as no-separation contact permitting frictionless tangential sliding; and surface-to-surface contact combined with bonded constraint was assigned between the articular processes and the corresponding vertebrae. The seven spinal ligaments (anterior longitudinal, posterior longitudinal, interspinous, supraspinous, intertransverse, ligamentum flavum, and facet capsular ligaments) were simulated using spring elements. All analyses were performed as linear static analyses in ANSYS Workbench (ANSYS 2021 R1, ANSYS, United States) on a Dell Precision 5,280 workstation (Intel Core i9-10940X @ 3.30 GHz, 64 GB RAM, NVIDIA GeForce RTX 4060, Windows 11 Pro 64-bit).

**FIGURE 3:**
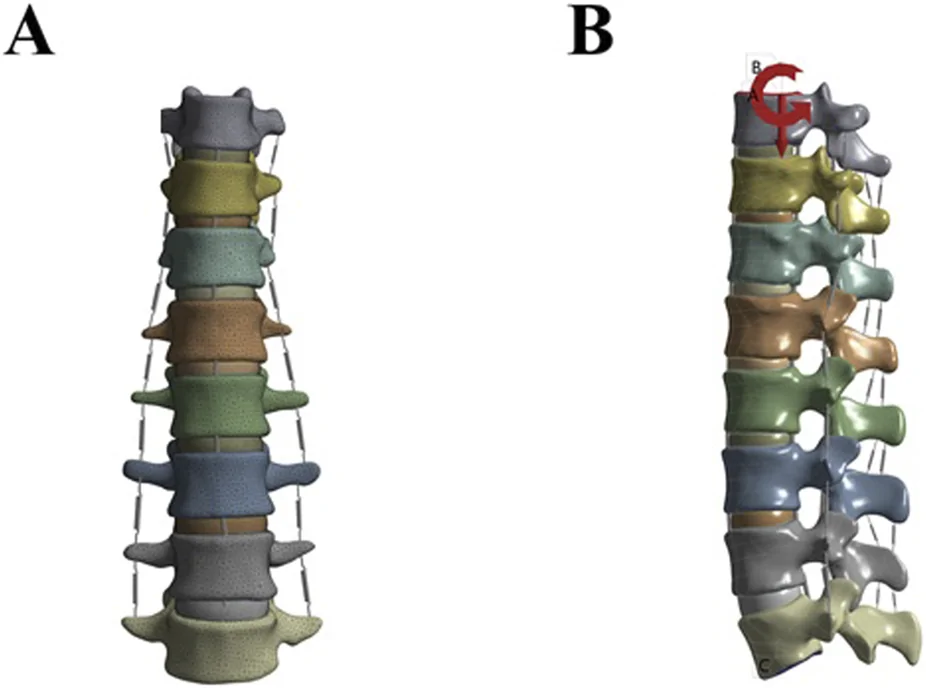
Using ANSYS software for mesh setup and loading setup. **(A)** Meshing the model. **(B)** Apply a 500 N vertical load on the superior endplate of T10 via a coupled reference node, along with 10 N·m moments in each direction (flexion, extension, lateral bending, and axial rotation) to simulate human movements.

### 2.5 Data analysis

Stress distribution in the T10 and T11 vertebrae and cancellous bone under the six motions was analyzed. The ROM of the T10-T11 segment under the six motions was observed. Stress contour maps under key loading conditions were compared among models to evaluate biomechanical changes under different conditions.

## 3 Results

### 3.1 Model validity verification

To validate the model, the inferior surface of L5 in Model A was constrained, and a 10 Nm torque was applied on the superior endplate of T10 in each direction to simulate six motions. Table 4 presents the ROM results for Model A. The ROM of Model A (normal control) under each motion was compared with previously reported experimental data for the lumbar spine (Yamamoto et al., 1989) and the thoracic spine (Wilke et al., 2017). The ROM of Model A showed good agreement with other studies, confirming the validity of the experimental model.

**TABLE 4:** The result of ROM data for Model A. Comparison of segmental ROM between Model A and previously reported *in vitro* data ((Li C. et al., 2024), 10 N·m, lumbar spine; Wilke et al. (Wang et al., 2023), thoracic spine, measured at ±7.5 N·m and normalized to 10 N·m).

Segment	Flexion	Extension	Left bending	Right bending	Left rotation	Right rotation
L1-L2 (Model A)	4.745	4.270	4.808	4.681	2.175	2.114
L1-L2 ((Yamamoto et al., 1989))	5.8	4.3	4.9	4.9	2.3	2.3
L2-L3 (Model A)	5.087	4.580	5.035	4.903	2.534	2.463
L2-L3 ((Yamamoto et al., 1989))	6.5	4.3	7.0	7.0	2.6	2.6
L3-L4 (Model A)	4.727	3.751	5.098	4.964	3.341	3.247
L3-L4 ((Yamamoto et al., 1989))	7.5	3.7	5.7	5.7	2.6	2.6
L4-L5 (Model A)	5.033	5.032	5.031	4.895	2.834	2.757
L4-L5 ((Yamamoto et al., 1989))	8.9	5.8	5.7	5.7	2.2	2.2
T10-T11 (Model A)	4.196	3.311	3.389	3.096	2.193	2.411
T10-T11 (Wang et al., 2025)*	4.5*	4.3*	5.4*	5.4*	5.5*	5.5*

By comparing the ROM data of previous literature, it is concluded that the ROM of Model A is in good agreement with the research results of other scholars, which proves the authenticity and effectiveness of this experimental model.

* Approximate values digitized from the published figures.

### 3.2 Range of motion analysis

Compared to the normal control group, the L4 spondylolisthesis model showed increased ROM of the T10-T11 segment in all six motions. During flexion, ROM increased from 4.196° to 4.384° (+4.5%); extension from 3.311° to 3.517° (+6.2%); left bending from 3.389° to 3.565° (+5.2%); right bending from 3.096° to 3.628° (+17.2%); left rotation from 2.193° to 2.543° (+16.0%); right rotation from 2.411° to 3.007° (+24.7%). The most pronounced increases were observed in rotation and right bending, suggesting that spondylolisthesis significantly affects rotational stability. The osteoporosis-only model showed even greater increases: flexion ROM reached 5.533° (+31.9% vs. normal), extension 3.602° (+8.8%), left bending 3.566° (+5.2%), right bending 3.657° (+18.1%), left rotation 3.467° (+58.1%), right rotation 3.806° (+57.9%). Notably, under rotational motions, osteoporosis led to a substantial increase in ROM, indicating a marked weakening of torsional stability. When spondylolisthesis and osteoporosis co-existed, ROM reached its maximum in all motions: flexion 5.686° (+35.5% vs. normal, +29.7% vs. spondylolisthesis alone, +2.8% vs. osteoporosis alone); extension 3.786° (+14.3% vs. normal, +7.6% vs. spondylolisthesis alone, +5.1% vs. osteoporosis alone); left bending 3.857° (+13.8% vs. normal, +8.2% vs. spondylolisthesis alone, +8.2% vs. osteoporosis alone); right bending 4.089° (+32.1% vs. normal, +12.7% vs. spondylolisthesis alone, +11.8% vs. osteoporosis alone); left rotation 3.777° (+72.2% vs. normal, +48.5% vs. spondylolisthesis alone, +8.9% vs. osteoporosis alone); right rotation 3.871° (+60.6% vs. normal, +28.7% vs. spondylolisthesis alone, +1.7% vs. osteoporosis alone).

Synergy analysis of ROM revealed a prominent synergistic effect during left lateral bending: the spondylolisthesis group increment was 5.19%, the osteoporosis group increment was 5.22%, yielding an arithmetic sum of 10.42%, whereas the combined group actual increment reached 13.81%, exceeding the additive sum by 3.39 percentage points (pp) and demonstrating a clear synergistic amplification effect. In flexion, extension, left lateral bending, and left rotation, the combined group increment was generally comparable to the sum of the individual increments, indicating predominantly additive effects, whereas during right rotation the combined increment (60.6%) was lower than the arithmetic sum of the individual increments (82.6%), suggesting a sub-additive interaction for this motion. Overall, the combined model exhibited ROM values higher than both normal and either single pathology alone in most motions, confirming cumulative destabilization (Figure 4).

**FIGURE 4:**
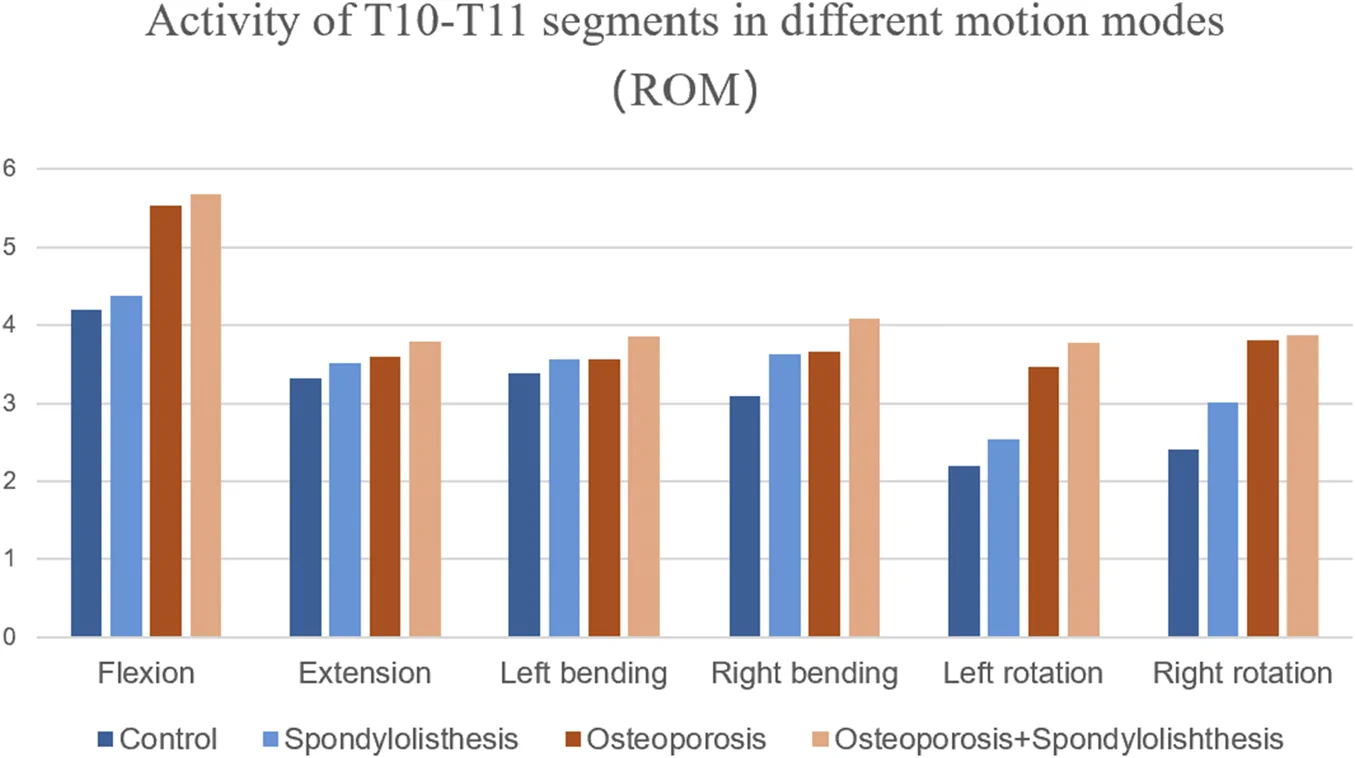
Results of the range of motion of the T10-T11 segment under different motion modes.

### 3.3 Vertebral body overall stress analysis

The overall maximum stress results for the T10 and T11 vertebrae are summarized in Figure 5. In the spondylolisthesis-only group, the isolated effect of spondylolisthesis led to modest increases in overall stress of T10 and T11, ranging from 0.4% to 15.1%. Overall and cancellous bone stresses of T10 and T11 were higher than normal in most motions, indicating that the stress redistribution caused by L4 spondylolisthesis extends to the thoracic region. The osteoporosis group showed substantially increased stress values. For example, during flexion, overall T10 stress increased from 33.42 MPa (normal) to 58.11 MPa, an increase of 73.9%; overall T11 stress increased from 28.79 MPa to 42.22 MPa, an increase of 46.6%. These increases far exceeded the changes in ROM, suggesting that osteoporosis has a more sensitive effect on vertebral stress. This directly reflects the mechanical principle that reduced bone material stiffness leads to significantly increased internal stress under the same external load. When both pathologies co-existed, stress peaked in all motions. T10 flexion stress rose to 59.07 MPa (+76.8% vs. normal), T11 to 43.34 MPa (+50.5% vs. normal); under lateral bending and extension, stress increases in the combined group generally exceeded 60%.

**FIGURE 5:**
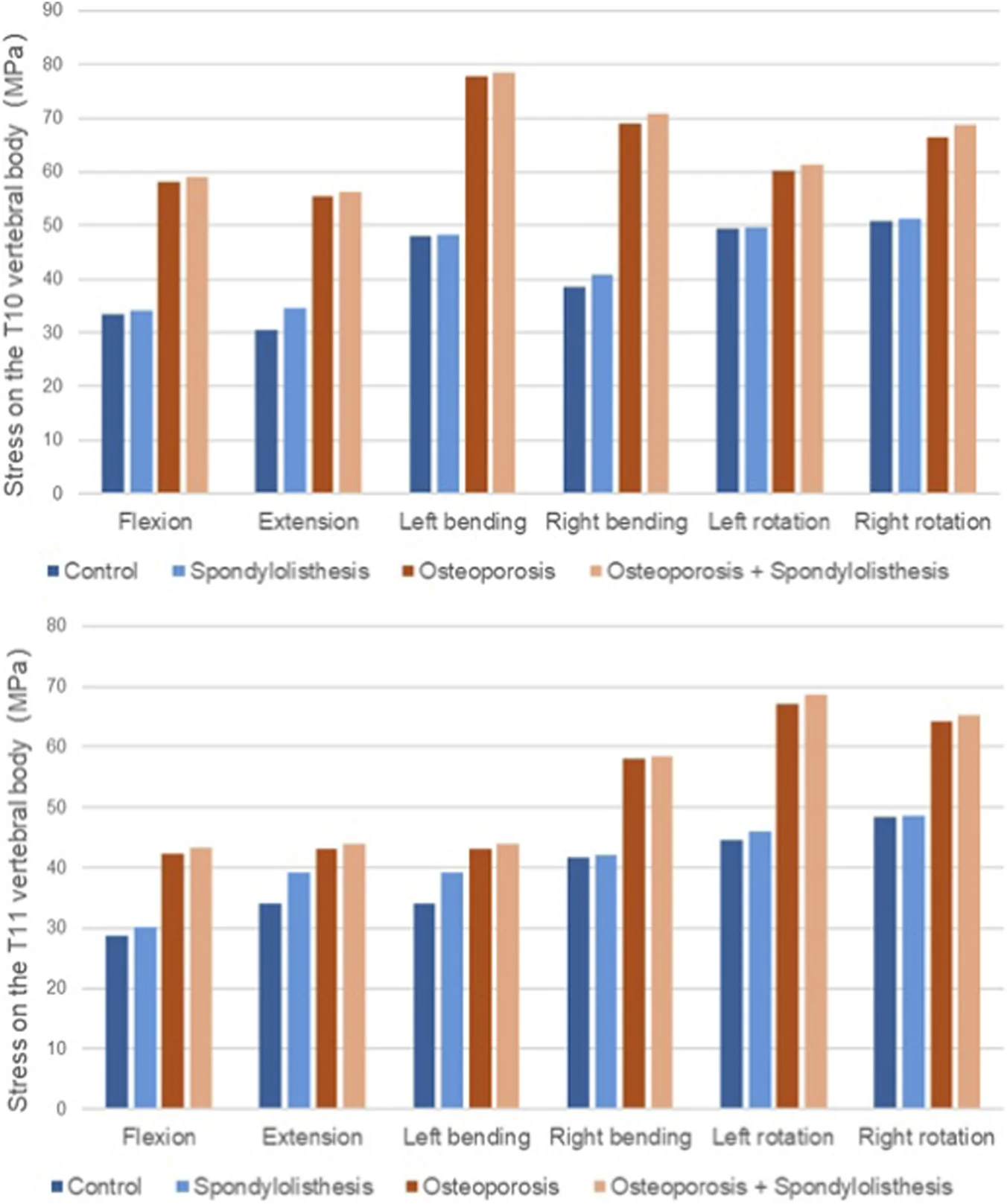
Overall maximum stress of the T10 and T11 vertebral bodies.

Synergy analysis revealed that the combined group increment exceeded the arithmetic sum of individual group increments in multiple motions, displaying a “1 + 1>2” synergistic amplification pattern. For T10 overall stress: during left lateral bending, the spondylolisthesis increment was 0.40%, the osteoporosis increment was 62.04%, arithmetic sum 62.44%, whereas the combined group actual increment was 63.49%, yielding a synergy of 1.05 pp; during left rotation, spondylolisthesis 0.57%, osteoporosis 22.02%, sum 22.59%, combined 24.62%, synergy 2.02 pp; during right rotation, spondylolisthesis 0.96%, osteoporosis 31.00%, sum 31.96%, combined 35.56%, synergy 3.61 pp. T11 overall stress showed similar trends: left rotation synergy 1.03 pp, right rotation synergy 1.44 pp. These data quantitatively confirm the mutual amplification of the two pathological factors in stress transmission.

### 3.4 Vertebral cancellous bone stress analysis

The maximum stress in cancellous bone is a key indicator for assessing fracture risk; data are shown in Figure 6. The trend in cancellous bone stress was similar to that of overall stress, but the effect of osteoporosis was more prominent. The spondylolisthesis group showed a slight increase in cancellous bone stress compared to normal, with increases <10% in most motions. The normal osteoporosis group showed significantly higher cancellous bone stress than the normal group; for example, for T10, flexion stress increased from 0.652 MPa to 0.780 MPa (+19.6%), extension stress from 0.470 MPa to 0.612 MPa (+30.2%). The spondylolisthesis + osteoporosis group reached the highest cancellous bone stress in nearly all motions. For instance, during flexion, T10 cancellous bone stress in Model D (0.833 MPa) was not only significantly higher than the control group but also higher than the spondylolisthesis-alone model (0.658 MPa) and the osteoporosis-alone model (0.780 MPa); T10 extension stress reached 0.654 MPa (+39.1% vs. normal), lateral bending stress reached 1.058 MPa (+28.6% vs. normal); T11 stresses showed similar increases.

**FIGURE 6:**
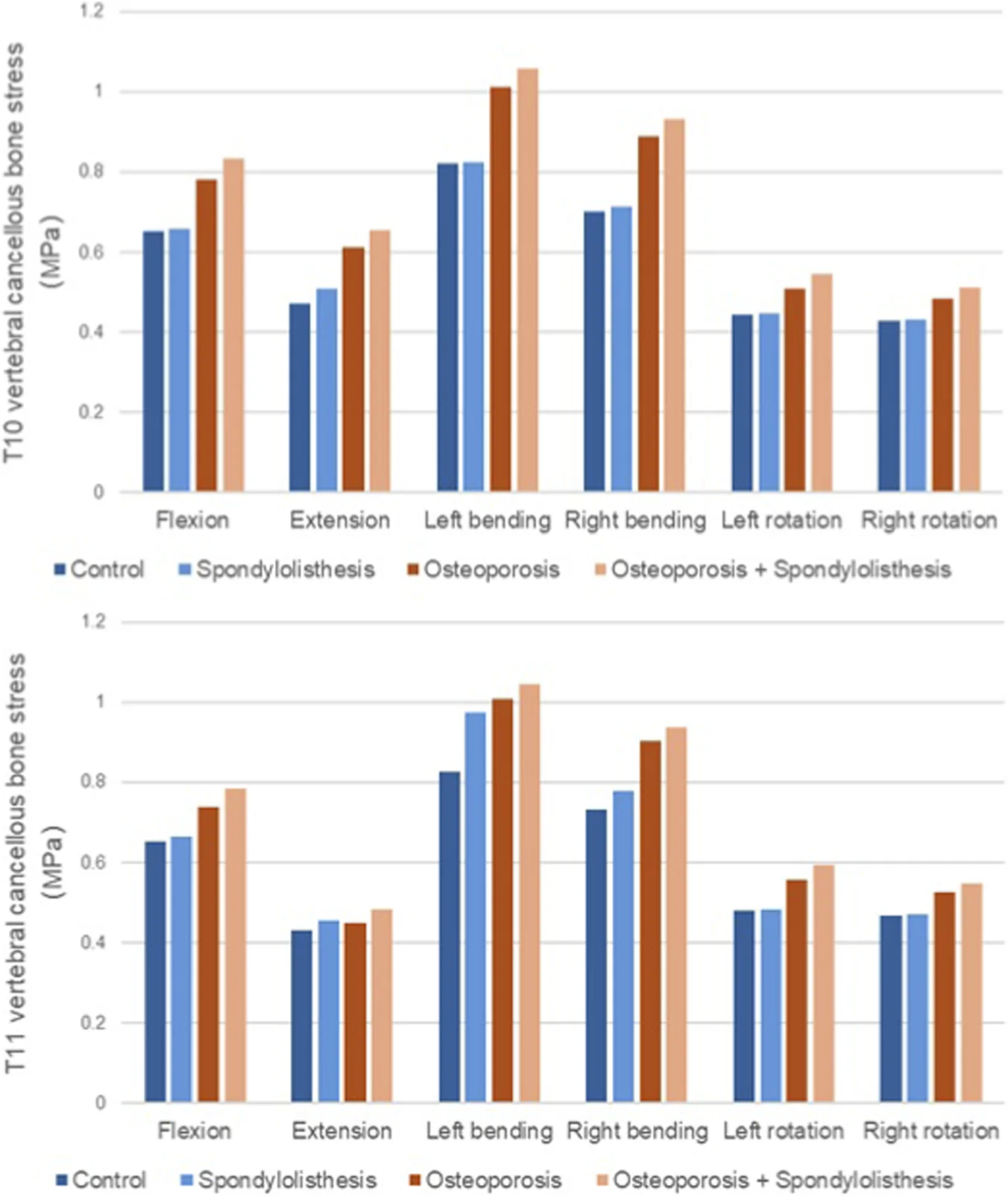
Maximum stress in the cancellous bone of the T10 and T11 vertebral bodies.

Quantitative synergy analysis further revealed even more pronounced synergistic amplification effects in cancellous bone stress. For T10 cancellous bone stress: during flexion, the spondylolisthesis increment was 1.06%, the osteoporosis increment was 19.73%, arithmetic sum 20.80%, combined group actual increment 27.85%, yielding a synergy of 7.05 pp; during extension, spondylolisthesis 8.04%, osteoporosis 30.25%, sum 38.29%, combined 39.14%, synergy 0.84 pp; during left lateral bending, spondylolisthesis 0.25%, osteoporosis 22.85%, sum 23.11%, combined 28.57%, synergy 5.46 pp; during right lateral bending, spondylolisthesis 1.98%, osteoporosis 26.96%, sum 28.94%, combined 33.14%, synergy 4.19 pp; during left rotation, spondylolisthesis 0.83%, osteoporosis 14.89%, sum 15.72%, combined 22.94%, synergy 7.23 pp; during right rotation, spondylolisthesis 0.55%, osteoporosis 13.33%, sum 13.88%, combined 19.38%, synergy 5.51 pp.

T11 cancellous bone stress also exhibited significant synergy: flexion 5.63 pp, left rotation 7.61 pp, right rotation 4.48 pp, extension 2.22 pp. These data demonstrate that cancellous bone, as the primary load-bearing structure of the vertebral body, exhibits the most prominent stress elevation under the combined action of both pathological factors, with synergistic effects generally exceeding those of overall stress, indicating that cancellous bone represents the critical target for fracture risk assessment.

### 3.5 Stress contour analysis

Stress contour maps under a representative motion visually display differences in stress distribution (in Figure 7). In all models, maximum stress was concentrated in the anterior and central regions of the T10 and T11 vertebral bodies and the pedicle regions, consistent with the clinically observed predilection sites for OVCFs. Compared to the normal control, the spondylolisthesis group showed an expanded high-stress area covering a larger anterior vertebral margin (in Figures 8, 9). The stress concentration was more pronounced in the osteoporosis-only group, with both the intensity and extent of high-stress areas significantly increased. The spondylolisthesis + osteoporosis group exhibited the most widespread distribution and highest intensity of stress concentration, with almost the entire anterior vertebral margin under high stress, indicating that the T10-T11 vertebrae are in the most unfavorable mechanical state in this model.

**FIGURE 7:**
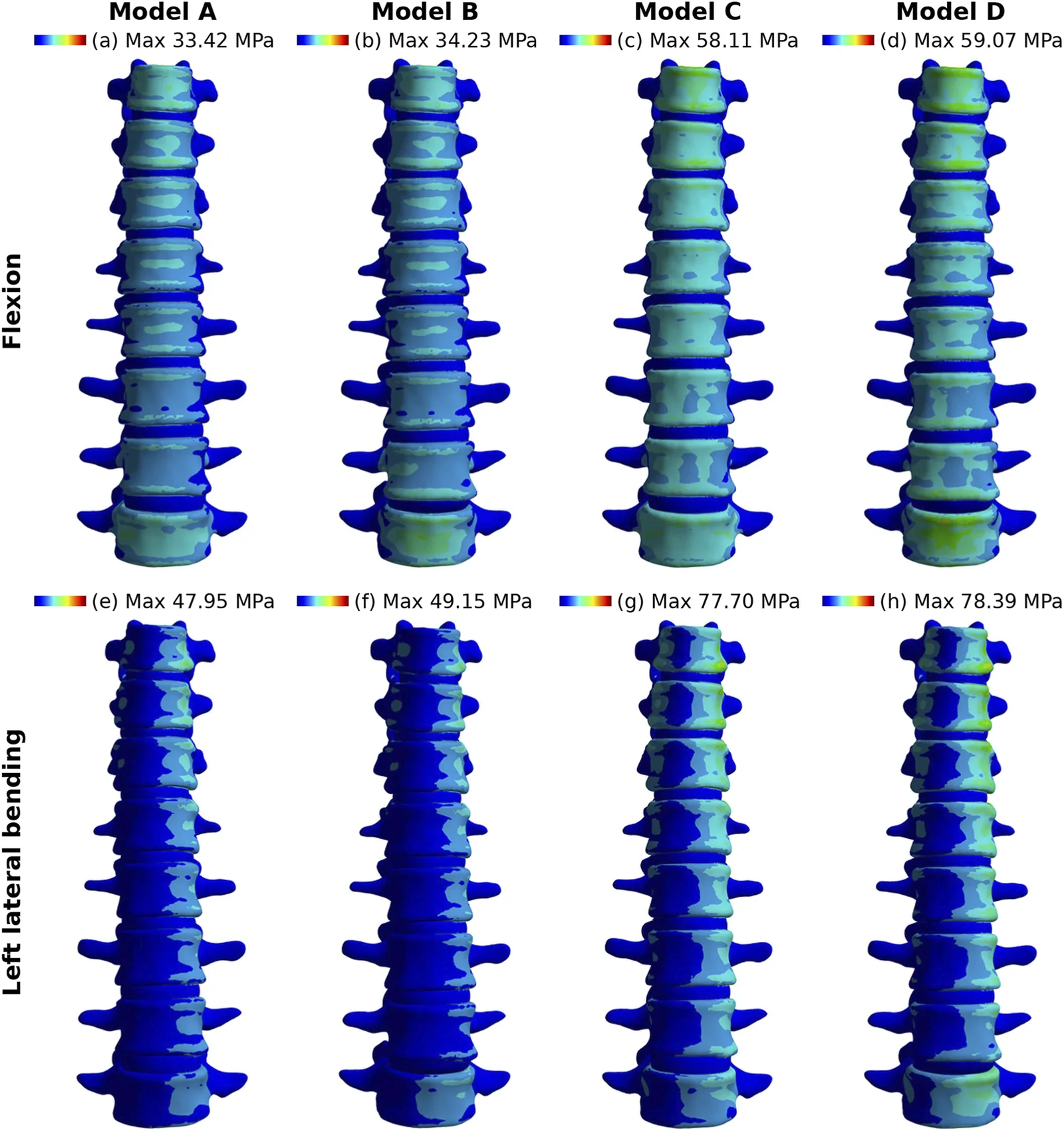
Equivalent (von Mises) stress contours of the whole spinal segment (T10–L5) in Models **(A–D)** under flexion **(a–d)** and left lateral bending **(e–h)**. The peak value (MPa) of each panel is indicated above its color scale.

**FIGURE 8:**
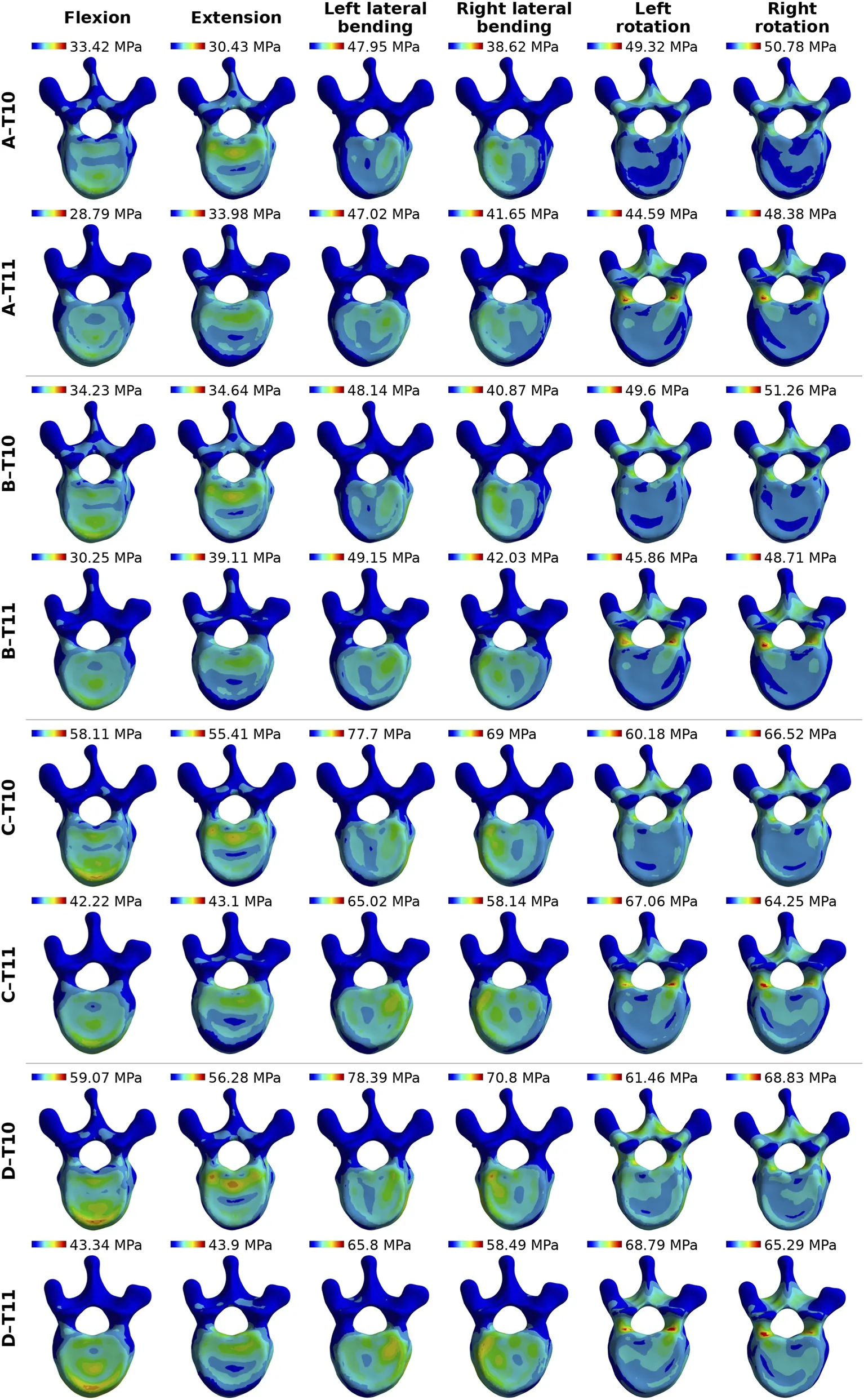
Equivalent (von Mises) stress contours of the whole T10 and T11 vertebrae (superior view) in Models A–D under six physiological loading conditions. The peak value (MPa) of each panel is indicated above its color scale.

**FIGURE 9:**
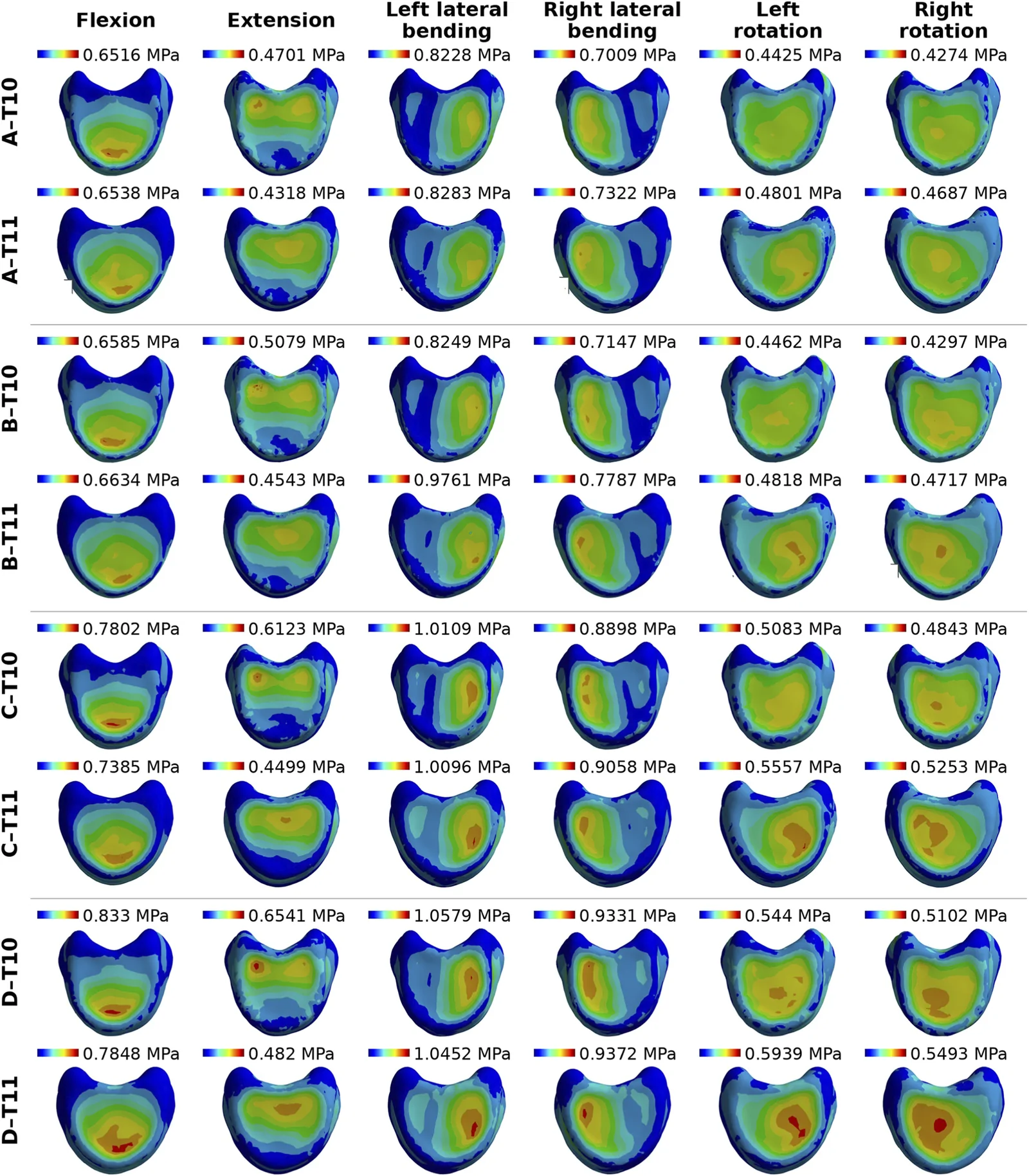
Equivalent (von Mises) stress contours of the cancellous bone of the T10 and T11 vertebrae (superior view) in Models A–D under six physiological loading conditions. The peak value (MPa) of each panel is indicated above its color scale.

To further illustrate the stress response under different physiological motions, von Mises stress contours of the T10 and T11 vertebrae were additionally examined under left and right lateral bending (Figure 10). Under left lateral bending, the peak von Mises stress of the normal model reached 47.95 MPa at T10 and 47.03 MPa at T11, whereas under right lateral bending the corresponding peaks were 38.62 MPa and 41.65 MPa, respectively. In both motions, high-stress regions were consistently concentrated in the anterior and central portions of the vertebral body and at the base of the pedicles, with a side-dependent redistribution toward the contralateral pedicle and posterolateral cortex, corroborating the flexion-based findings and indicating that the stress concentration pattern at T10-T11 is consistent across physiological motions.

**FIGURE 10:**
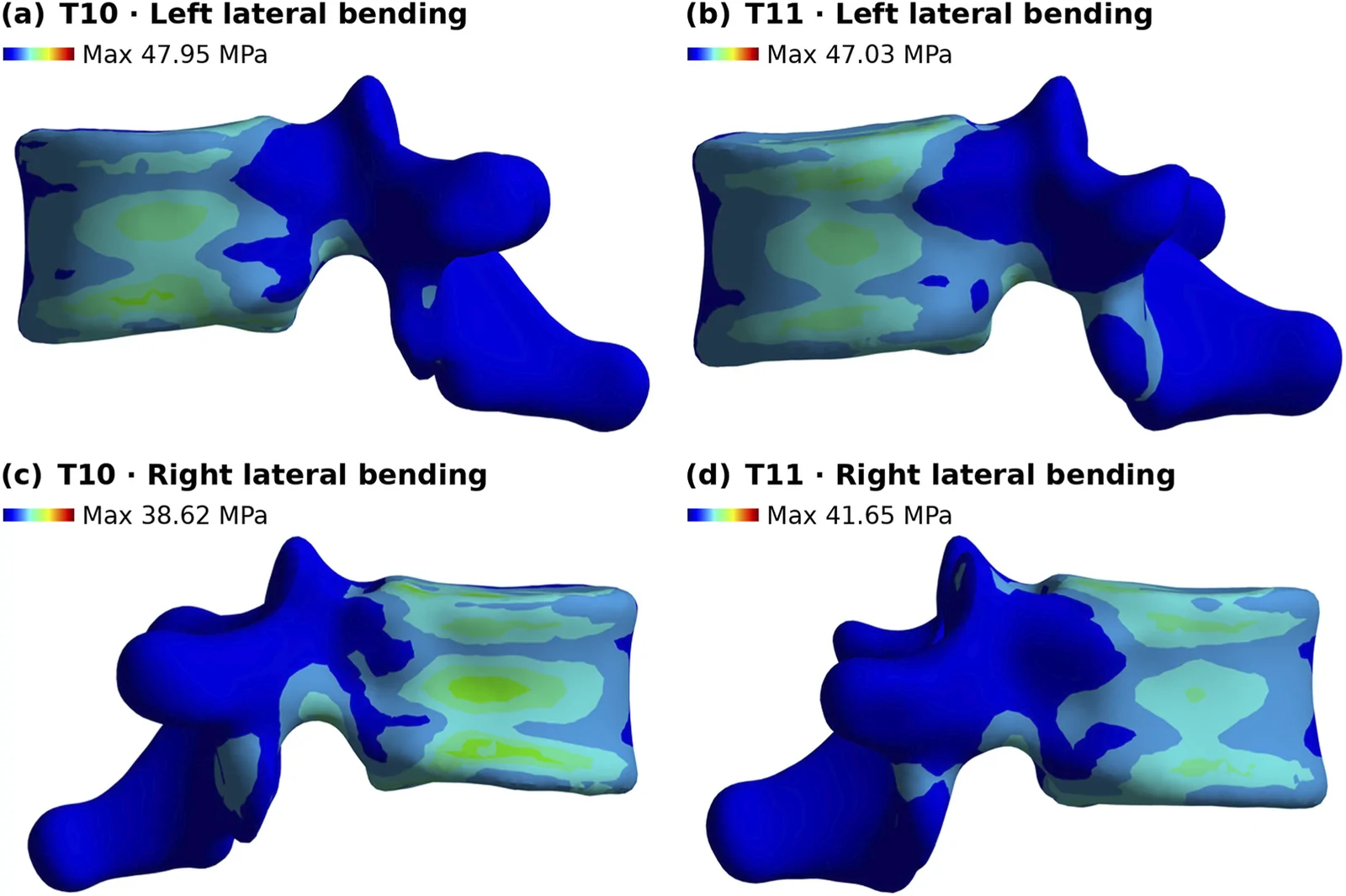
Stress contours (lateral view) of the T10 and T11 vertebrae of the normal model under left and right lateral bending: **(a)** T10 under left lateral bending; **(b)** T11 under left lateral bending; **(c)** T10 under right lateral bending; **(d)** T11 under right lateral bending.

Total deformation of the entire T10–L5 segment under flexion is shown in Figure 11. In all four models, the displacement increased smoothly and monotonically from the fully constrained inferior surface of L5 (0 mm) toward the cranial end of T10, without any abrupt local discontinuity, indicating a physiologically consistent deformation pattern. The maximum deformation occurred at the superior aspect of T10, reaching 82.99, 86.82, 87.80, and 101.85 mm in Models A, B, C, and D, respectively. Notably, the combined model (Model D) exhibited the largest deformation, consistent with the superimposed destabilizing effect of L4 spondylolisthesis on osteoporotic bone. It should be emphasized that these values represent the cumulative displacement at the cranial end of the whole spinal column; the corresponding segmental ROM remained only a few degrees (Table 5), confirming that the model operates within the physiological motion range.

**FIGURE 11:**
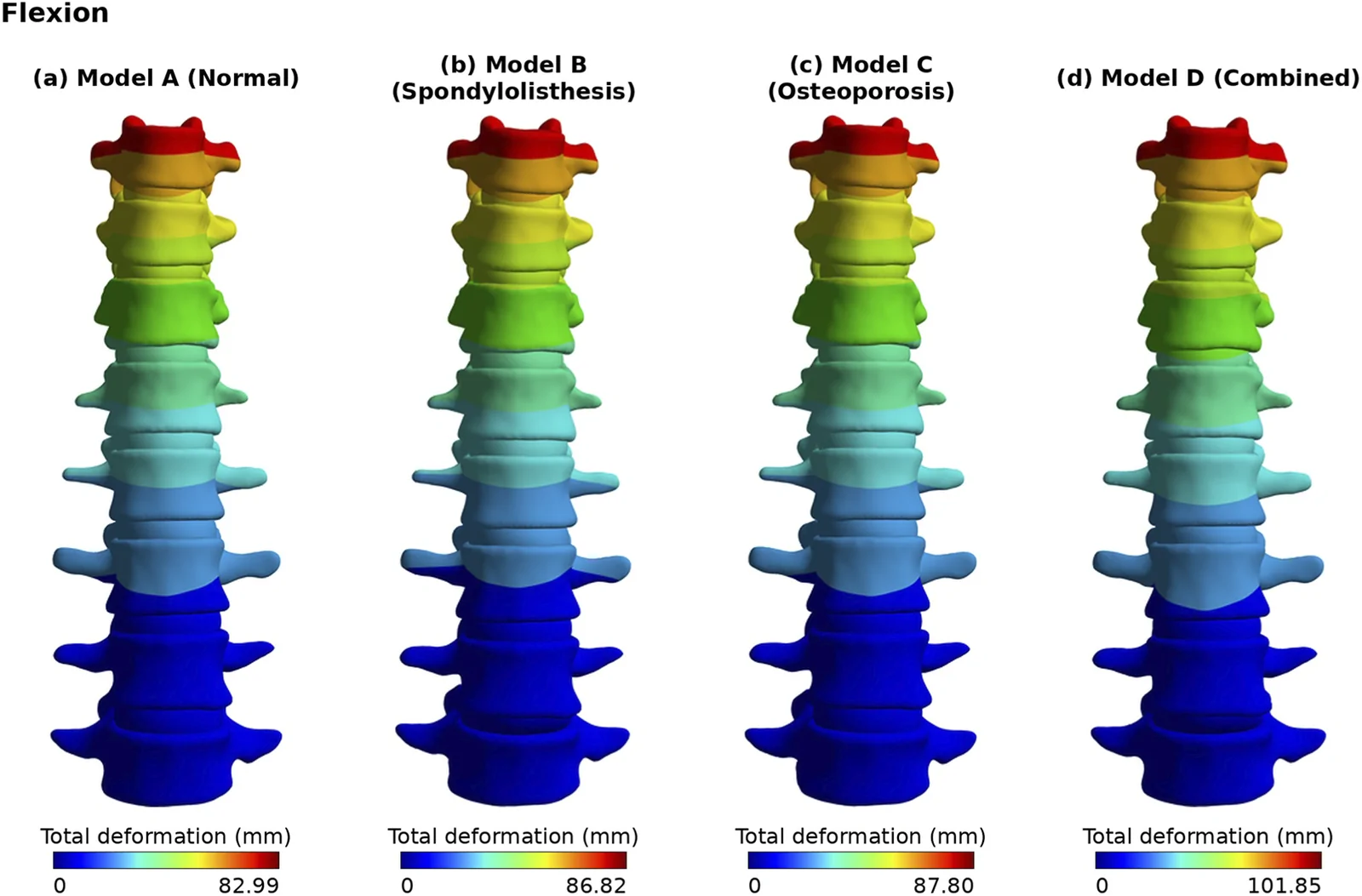
Total deformation contours of the T10–L5 spinal segment under flexion in the four models: **(a)** Model **(A)** (normal); **(b)** Model **(B)** (spondylolisthesis); **(c)** Model **(C)** (osteoporosis); **(d)** Model **(D)** (spondylolisthesis combined with osteoporosis). The inferior surface of L5 was fully constrained (blue, 0 mm), and the maximum deformation occurred at the cranial end of T10 (red). The maximum value of each model is indicated at the right end of the corresponding color bar. Unit: mm.

**TABLE 5:** Range of motion (ROM, °) of the T10–T11 segment and relative displacement (mm) of the L4 vertebra in the four models under six loading conditions.

Loading condition	Model A (normal)	Model B (spondylolisthesis)	Model C (osteoporosis)	Model D (combined)
(a) ROM of the T10–T11 segment (°)				
Flexion	4.20	4.38	5.53	5.69
Extension	3.31	3.52	3.60	3.79
Left lateral bending	3.39	3.57	3.57	3.86
Right lateral bending	3.10	3.63	3.66	4.09
Left rotation	2.19	2.54	3.47	3.78
Right rotation	2.41	3.01	3.81	3.87

Loading condition	Model A (Normal)	Model B (Spondylolisthesis)	Model C (Osteoporosis)	Model D (Combined)
(b) Relative displacement of the L4 vertebra (mm)				
Flexion	6.61	7.58	7.08	8.21
Extension	4.05	3.71	4.43	4.12
Left lateral bending	3.96	4.16	4.35	4.43
Right lateral bending	5.03	4.68	5.31	5.04
Left rotation	3.43	8.54	3.48	8.83
Right rotation	3.66	8.20	3.88	8.44

Values are reported to two decimal places. ROM was defined as the angular motion of the superior endplate of T10 relative to the inferior endplate of T11; L4 displacement was defined as the resultant displacement of the L4 vertebral body relative to the fully constrained inferior surface of L5.

## 4 Discussion

This study, by constructing detailed three-dimensional finite element models of the spine, systematically analyzed for the first time the biomechanical effects of L4 spondylolisthesis and osteoporosis on non-adjacent thoracic segments (T10-T11). The results show that even when spondylolisthesis occurs at L4, lumbar spondylolisthesis still leads to significantly increased range of motion and stress in the superior T10-T11 segment; osteoporosis directly weakens the structural stiffness of the vertebrae, causing a dramatic increase in internal stress; when both coexist, their negative effects are mutually amplified, which may render the thoracic spine more susceptible to osteoporotic compression fractures.

Lumbar spondylolisthesis, particularly degenerative lumbar spondylolisthesis, is a common spinal disorder. Its pathophysiological basis is degeneration of the intervertebral disc and facet joints, leading to anterior or posterior vertebral displacement (Enyo et al., 2015). From a biomechanical perspective, lumbar spondylolisthesis disrupts the normal sagittal balance of the spine, causing a deviation in load transfer pathways (Hani et al., 2025). To maintain overall sagittal balance, the thoracolumbar junction (e.g., T10-T11) may undergo a series of compensations, resulting in locally increased stress and altered motion patterns (Bruno et al., 2012). Our data support this view: the spondylolisthesis group showed particularly increased ROM in right bending and rotation. Increased ROM implies decreased segmental stability, which predicts an increased risk of degeneration and injury. This decrease in stability affects not only the slipped segment itself but also adjacent and even distant segments (Li K. et al., 2024; Zhao et al., 2023), creating a so-called “remote effect”. This “remote effect” explains why some patients with lumbar spondylolisthesis, even without severe lumbar symptoms, may develop thoracic back pain or thoracic vertebral fractures. This finding suggests that abnormal upward transmission of biomechanical load is an important mechanism (Okamoto et al., 2015), capable of affecting even distant regions. Clinically, OVCFs most frequently cluster at the T12-L1 thoracolumbar junction, and the T10-T11 segments examined in this study lie immediately cranial to this high-incidence region and represent the segments most remote from the L4 lesion.

Osteoporosis reduces bone mineral density and destroys bone microarchitecture, significantly decreasing the load-bearing capacity of vertebrae (Ling et al., 2025). As expected, the osteoporosis model (normal osteoporosis group) exhibited the highest overall and cancellous bone stresses under nearly all motions, with increases far exceeding those due to spondylolisthesis alone. According to basic mechanics of materials principles, the sharp decrease in bone elastic modulus caused by osteoporosis is the fundamental reason for the substantial increase in vertebral stress (especially cancellous bone stress, which determines fracture risk) in Model C (Wang et al., 2023). This study quantified this effect: for example, under flexion, osteoporosis increased T10 overall stress from 33.42 MPa to 58.11 MPa, an increase of 74%, which far exceeded the change in ROM (31.9%), suggesting that osteoporosis has a more sensitive effect on vertebral stress. More importantly, cancellous bone, as the main load-bearing and metabolically active part of the vertebra, has stress levels that are key indicators for predicting fracture risk. This study shows that under osteoporotic conditions, cancellous bone stress in T10 and T11 generally increases by 12%–30%, meaning that the mechanical demand on trabeculae already weakened by osteoporosis is substantially increased, which may contribute to a mechanically less favourable environment for the vertebrae. From a microscopic perspective, stress concentration regions in osteoporotic trabeculae are more prone to microfractures, and the accumulation of microfractures eventually leads to macroscopic fractures, consistent with our final stress contour results (Broy, 2016).

One of the most clinically significant findings of this study is that the synergistic effect of spondylolisthesis and osteoporosis is not a simple addition; the results of Model D are not the arithmetic sum of Models B and C but exhibit a “1 + 1>2” amplification pattern. Quantitative analysis showed that among 17 motion-metric combinations, 16 exhibited positive synergy (synergy >0), with 12 showing synergy magnitude exceeding 1 percentage point.

Cancellous bone stress showed the most prominent synergistic effects. For T10 cancellous bone: flexion synergy 7.05 pp, left rotation 7.23 pp, right rotation 5.51 pp. For T11 cancellous bone: left rotation 7.61 pp, flexion 5.63 pp. Overall vertebral stress also demonstrated significant synergy: right rotation T10 + 3.61 pp, left rotation T10 + 2.02 pp, right rotation T11 + 1.44 pp. ROM showed synergy only during left lateral bending (3.39 pp), with additive effects predominating in other motions.

This “1 + 1>2” synergistic pattern can be explained mechanistically as follows: spondylolisthesis alters spinal load transfer, bringing abnormal stress changes to T10-T11; meanwhile, osteoporosis substantially reduces the intrinsic load-bearing capacity and structural stiffness of the vertebrae at that segment (Ling et al., 2025; Wang et al., 2023). When both coexist, the abnormal mechanical load caused by slippage acts on vertebral structures already weakened by osteoporosis, resulting in stress elevation far exceeding the linear superposition of individual effects. The reason cancellous bone exhibits the most significant synergy is that, as the primary load-bearing and metabolically active component of the vertebra, its trabeculae become sparse and the elastic modulus significantly decreases under osteoporotic conditions, while the additional shear and torsional loads from spondylolisthesis subject the trabeculae to more complex stress states, creating a biomechanical “vicious cycle.” This finding provides a precise quantitative biomechanical explanation for previous retrospective clinical findings that “lumbar spondylolisthesis is an independent risk factor for thoracolumbar OVCFs” (Wang et al., 2022; Wang et al., 2018), consistent with the synergistic phenomenon reported by Wang et al. (Wang et al., 2025).

It should be emphasized that, although spondylolisthesis and osteoporosis exhibited a synergistic interaction, the magnitude of their individual contributions differed markedly: the isolated effect of L4 spondylolisthesis on T10-T11 stress was only 0.4%–15.1%, whereas osteoporosis alone increased T10 overall stress by up to 73.9% and dominated the stress elevation observed in the combined model. Osteoporosis should therefore be regarded as the principal determinant of T10-T11 vertebral stress, with spondylolisthesis acting as a secondary biomechanical modifier. Nevertheless, the clinical relevance of spondylolisthesis lies less in its isolated effect than in its synergistic interaction with osteoporosis: the synergy was most prominent in cancellous bone (up to 7.05 and 7.61 percentage points for T10 and T11, respectively, far exceeding the synergy observed for overall stress and ROM), indicating that once vertebrae are weakened by osteoporosis, the additional mechanical disturbance introduced by spondylolisthesis is disproportionately amplified precisely in the structure that governs fracture initiation. This finding appears to contrast with previous epidemiological studies, including our own, which identified lumbar spondylolisthesis as an independent risk factor for thoracolumbar OVCFs (Wang et al., 2022; Wang et al., 2018). Several considerations may reconcile this apparent discrepancy. First, finite element analysis isolates individual factors under otherwise identical conditions, whereas in clinical cohorts spondylolisthesis frequently coexists with intervertebral disc degeneration, sagittal imbalance, and altered muscle function, none of which were simulated here; the clinical risk attributed to spondylolisthesis may partly reflect these accompanying pathologies. Second, spondylolisthesis may serve as a marker of global spinal degeneration that parallels, rather than directly causes, the deterioration of vertebral strength. Third, although the acute biomechanical effect of spondylolisthesis on thoracic stress is modest, the associated increase in segmental motion and the resulting chronic instability may accumulate over years and thereby contribute to fracture risk in osteoporotic vertebrae. These interpretations warrant verification in combined biomechanical and prospective clinical studies.

The results of this study have important implications for clinical practice. First, the evaluation of osteoporotic patients should not be limited to bone mineral density measurement. Especially during spinal imaging examinations, the presence of lumbar spondylolisthesis should be routinely assessed. Even asymptomatic spondylolisthesis should be regarded as an important risk marker, indicating that the patient may have a higher risk of thoracic vertebral fractures. Second, for osteoporotic patients with concurrent lumbar spondylolisthesis, more aggressive interventions should be taken. In addition to standard intensified anti-osteoporosis treatment, rehabilitation measures such as core muscle strength training and postural correction may be needed to enhance spinal stability. Third, for patients with progressive spondylolisthesis, severe sagittal imbalance, or refractory pain, the necessity and timing of surgical intervention should be evaluated. Stabilizing the slipped segment and restoring normal spinal curvature may help prevent secondary thoracolumbar vertebral fractures.

This study systematically analyzed for the first time the biomechanical effects of L4 spondylolisthesis and osteoporosis on non-adjacent thoracic segments (T10-T11) and achieved certain results. However, some limitations remain that need to be addressed in future research:

First, the material model adopted linear elastic assumptions. Although suitable for predicting stress distribution before yield, it cannot simulate plastic deformation, microcrack propagation, or the eventual fracture process. Future studies may incorporate damage mechanics or cohesive zone models. In addition, a simplified 500 N vertical compressive load was applied instead of a follower load, and muscle forces were not simulated; although this simplification is common in comparable finite element studies and affects all four models equally, future work should incorporate follower-load protocols to better replicate physiological spinal loading. Second, this study did not consider individual factors such as muscle forces or disc degeneration, which might alter the absolute values of ROM and stress to some extent. However, given that this study focuses on comparisons between different models, the impact of this simplification on the main conclusions is limited. Future research could introduce more complex physiological loading and degeneration models. Third, this study simulated only one degree of spondylolisthesis and did not explore the differential effects of various grades and types. Future studies will analyze the effects of different spondylolisthesis severities and seek validation through *in vitro* biomechanical experiments. Finally, although the model validity was confirmed by comparison with literature data, direct validation of the predicted results through matched *in vitro* biomechanical experiments will be an important next step to further improve the predictive accuracy and clinical applicability of the model.

## 5 Conclusion

This study successfully established and validated four finite element models of the lumbar spine with distinct characteristics (spondylolisthesis, osteoporosis). The biomechanical analysis revealed that L4 spondylolisthesis alone reduces spinal stability, increasing vertebral stress and range of motion at T10-T11. Osteoporosis alone exacerbates these changes, particularly in cancellous bone, thereby increasing fracture risk. When both conditions coexist, a synergistic amplification effect emerges, superimposing increased mechanical load onto compromised structural integrity, which may increase the biomechanical susceptibility to secondary osteoporotic compression fractures. These findings elucidate the mechanical basis for lumbar spondylolisthesis as a risk factor for thoracic vertebral fractures, providing an important theoretical foundation for clinical risk identification, etiological understanding, and comprehensive prevention strategies.

## Data Availability

The original contributions presented in the study are included in the article/supplementary material, further inquiries can be directed to the corresponding author.
